# Comprehensive analysis of calcification frequency and patterns in ovarian tumours using non-contrast CT

**DOI:** 10.1007/s11604-025-01750-4

**Published:** 2025-03-05

**Authors:** Tsukasa Saida, Miki Yoshida, Saki Shibuki, Toshitaka Ishiguro, Sodai Hoshiai, Masafumi Sakai, Taishi Amano, Ayumi Shikama, Toyomi Satoh, Takahito Nakajima

**Affiliations:** 1 Department of Radiology, Institute of Medicine, University of Tsukuba, 1-1-1 Tennodai, Tsukuba, Ibaraki J305-8575 Japan; 2 Department of Diagnostic and Interventional Radiology, University of Tsukuba Hospital, 2-1-1 Amakubo, Tsukuba, Ibaraki 305-8576 Japan; 3 Department of Obstetrics and Gynecology, Institute of Medicine, University of Tsukuba, 1-1-1 Tennodai, Tsukuba, Ibaraki 305-8575 Japan

**Keywords:** Ovary, Tumour, Computed tomography, Calcification

## Abstract

**Objectives:**

To investigate the frequency and patterns of calcification in ovarian tumours and evaluate their association with various histological types and malignancy grades.

**Methods:**

This retrospective study included patients who underwent non-contrast CT between March 2015 and March 2024 and had pathologically confirmed ovarian tumours. CT scans were reviewed for the presence and patterns of calcification (punctate, linear, coarse, and amorphous) by three radiologists. Statistical analysis was performed using the Fisher–Freeman–Halton exact test with Bonferroni correction.

**Results:**

This study included 328 patients (mean age, 55 years; range, 18–88 years). Significant differences in calcification frequency were observed among major tumour categories (*p* < 0.001), with with germ cell tumours being more calcified and metastases less calcified. Similarly, a significant difference was also found among epithelial tumours (*p* = 0.005), where mucinous and Brenner tumours were more calcified, whereas serous tumours were less calcified. Benign epithelial tumours showed a significantly higher frequency of calcification than borderline tumours and carcinomas (*p* < 0.001). When comparing the calcification patterns observed among epithelial tumours, significant differences were found for all calcification patterns: punctate (*p* = 0.024), linear (*p* < 0.001), coarse (*p* < 0.001), and amorphous (*p* < 0.001). The linear pattern was more common in mucinous tumours, whereas the amorphous pattern was more common in serous and Brenner tumours. Among non-epithelial tumours, germ cell tumours frequently exhibited liner and many calcifications, and immature teratomas were characterised by a mixture of punctate, linear, and coarse calcifications. Granulosa cells and metastatic tumours did not exhibit calcification.

**Conclusions:**

Among epithelial tumours, mucinous and Brenner tumours had a significantly higher frequency of calcification, and benign tumours had a significantly higher frequency of calcification. Amorphous patterns were significantly more common in serous and Brenner tumours, while linear patterns were significantly more common in mucinous tumours.

## Introduction

Ovarian tumours encompass a diverse group of neoplasms with varying histological characteristics, clinical behaviours, and prognostic implications. Among these, calcification is a noteworthy feature frequently observed in various types of ovarian tumours and appears in different patterns, including psammomatous and stromal calcifications. For instance, psammoma bodies are commonly associated with serous carcinomas [1], whereas stromal calcification may indicate tumours rich in fibrous stroma, such as fibromas [2]. The identification and interpretation of these calcifications can aid in diagnosing specific histological types and distinguishing between benign and malignant, thus influencing clinical decision-making and patient management. Despite their potential importance, previous studies have primarily focused on specific types of ovarian tumours [3–11], and frequency and the patterns of calcification in ovarian tumours have not been comprehensively analysed in large cohorts. In addition, many previous validations have included evaluation with contrast-enhanced CT [3–10], which may not accurately detect faint calcifications, and non-contrast CT is more appropriate for accurate assessment of calcification. In this study, we aimed to perform a thorough analysis of ovarian tumours using non-contrast CT to determine the frequency and patterns of calcification and to provide a detailed characterisation of calcification and its association with various histological types. Our findings are expected to enhance our understanding of calcification in ovarian tumours and contribute to improved diagnostic accuracy.

## Materials and methods

### Patients

The protocol for this retrospective study was approved by our Institutional Review Board (approval number: R05-240), which waived the requirement for written informed consent, owing to the retrospective nature of this study.

**Fig. 1:**
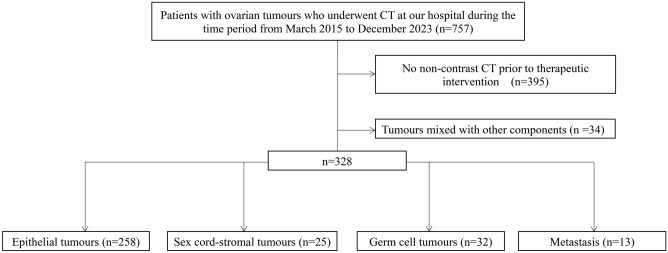
Flowchart of the patient selection process

The inclusion criteria were as follows: (a) women aged > 18 years (for ethical reasons); (b) patients who had undergone ovarian tumour resection and had pathologically confirmed ovarian tumours at our hospital, identified through the electronic medical record system; and (c) patients who underwent CT scans and MRI at our hospital between March 2015 and March 2024, identified through the Radiology Information System. We excluded the following cases: (a) patients with tumours mixed with other components; and (b) patients who had not undergone non-contrast CT prior to therapeutic intervention. All eligible patients who fulfilled the inclusion.

criteria and did not meet any of the exclusion criteria were included in the study. In cases of bilateral tumours, only the larger one was targeted. A flowchart of the patient selection process is shown in Fig. 1.

### CT examination

At our institution, when a malignancy is suspected, we perform contrast-enhanced CT scans to investigate metastasis; if an ovarian tumour is suspected, an additional non-contrast CT scan of the tumour site is conducted to evaluate the presence of enhancement by comparing the images before and after contrast administration. Therefore, if the initial MRI confirmed a benign tumour, or if a CT scan performed at a previous facility could be used as a substitute, the CT scan was omitted. Additionally, there were instances in which a non-contrast CT scan was not performed, such as when tumours were incidentally discovered on contrast-enhanced CT scans. CT scans were performed using a Brilliance 64, iCT256, or IQon spectral CT scanner (Philips Medical Systems, Amsterdam, Netherlands) with a tube voltage of 120 kVp and 100 to 220 mA current, and the images to be evaluated had a slice thickness of 2 mm.

### Clinical and pathological findings

The age at onset and pathological diagnosis were obtained from the electronic medical record system. Histological diagnosis was based on the WHO classification at the time of pathological diagnosis. The laterality and malignancy grades of the tumours were also recorded simultaneously.

### Image analysis

**Fig. 2:**
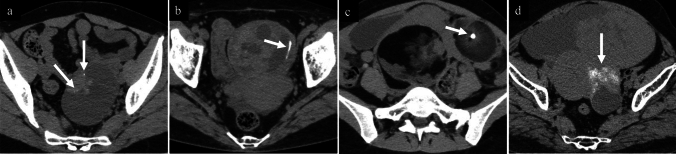
Patterns of calcification: **a**; Punctate (Mucinous carcinoma), **b**; Linear (Mucinous cystadenoma), **c**; Coarse (Mature teratoma), **d**; Amorphous (Brenner tumour)

The images in the present study were reviewed by three radiologists with 16, 4, and 4 years of postcertification experience specialising in gynaecologic imaging. They were unaware of the clinical and pathological findings of each patient and independently reviewed the images using the Centricity Universal Viewer (GE Healthcare, Chicago, IL, United States) with freely changed window levels and widths, in case of disagreement, discussions were held, and the final decisions were made through consensus. Tumours with even a single calcification inside or at the periphery of the primary tumour were categorised as having calcification. If calcification was present, the pattern was classified as punctate, linear, coarse, or amorphous. Punctate calcification was defined as round calcification ≤ 2 mm, with a density of ≥ 130 HU. Linear calcification was defined as elongated calcification with a short axis of ≤ 2 mm, with a density of ≥ 130 HU. Coarse calcification was defined as dense calcification > 2 mm, with a density of ≥ 130 HU. Amorphous calcification was defined as indistinct calcification that appeared powdery, cloudy, or cotton-like, with a density of < 130 HU. Representative CT images of these calcification patterns are shown in Fig. 2. When multiple calcification patterns coexisted, each pattern was recorded. The number of calcifications within a single tumour was counted and classified as few if there were 9 or fewer, and many if there were 10 or more. The distribution of the calcifications was evaluated to determine whether they were focal or diffuse, and diffuse calcification was defined as calcifications distributed evenly throughout the entire tumour. The localisation of the calcifications was evaluated whether they were located on the cyst wall, septa, or in the solid tissue [12]. In solid and cystic tumours, if calcifications were present in both the cyst wall and the solid tissue, they were registered in both locations. When it was difficult to identify the tumour using non-contrast CT alone, contrast-enhanced CT or MRI scans were referenced to determine the location of the target tumour.

### Statistical analysis

The presence and patterns of calcifications were compared among epithelial tumours, sex cord-stromal tumours, germ cell tumours, and metastases. These were also compared between different histological types of epithelial tumours and by grade of malignancy (benign tumour, borderline tumour, carcinoma) for all epithelial tumours. The Fisher–Freeman–Halton exact test with Bonferroni correction was used for these comparisons. The Fisher–Freeman–Halton exact test is a nonparametric test used for qualitative data with ≥ 3 groups. Bonferroni correction is a method used to adjust the significance level of multiple comparisons to reduce the probability of a type I error.

The agreement of the three readers was assessed using Fleiss’ kappa (κ) statistics. The κ-statistic interpreted the agreement as follows: < 0, no; 0–0.20, slight; 0.21–0.40, fair; 0.41–0.60, moderate; 0.61–0.80, substantial; and 0.81–1.00, almost perfect.

All statistical analyses were performed using SPSS software (SPSS Statistics 29.0; IBM, New York, NY, USA), and statistical significance was set at *p* < 0.05.

## Results

This study included 328 patients (mean age, 55 years; range, 18–88 years). A summary of the demographic characteristics of this study is presented in Tables 1, and 2 shows the number of cases, frequency of calcification, frequency of each calcification pattern, number of calcifications, and their distribution and localisation across all types of ovarian tumours, while Table 3 focuses on these parameters specifically across epithelial tumours.

**Table 1:** A summary of the demographic characteristics

Pathology	Number of cases	Mean age	Slandered deviation of age	Age range
Epithelial tumour	258	56	15	18–87
Serous tumour	85	57	14	25–87
Mucinous tumour	70	53	18	18–84
Clear cell tumour	57	56	15	32–84
Endometrioid tumour	29	58	14	30–79
Seromucinous tumour	12	55	18	33–81
Brenner tumour	5	74	3	68–77
Sex cord-stromal tumour	25	55	18	21–88
Fibroma	13	55	20	21–88
Thecoma	2	59	13	46–72
Granulosa cell tumour	10	54	14	31–77
Germ cell tumour	32	44	19	18–87
Mature teratoma	15	53	18	22–87
Immature teratoma	3	19	8	18–27
Struma ovarii	10	48	12	25–60
York sac tumour	3	26	7	18–32
Dysgerminoma	1	18	0	18
Metastasis	13	52	12	28–70

**Table 2:** The number of cases, frequency of calcification, frequency of each calcification pattern, number of calcifications, and their distribution and localisation across all types of ovarian tumours

Pathology	Cases (n)	Calcification (%)	Punctate/Linear/Coarse/Amorphous (%)	Few/Many (%)	Focal/Diffuse (%)	Cyst wall or septa/Solid part (%)
Epithelial tumour	258	35	27/15/5/10	22/13	30/5	28/9
Benign	40	63	43/30/25/15	35/28	55/8	53/10
BLT	44	32	30/16/7/7	18/14	27/5	32/0
Carcinoma	174	31	22/11/5/10	21/10	25/6	22/11
Sex cord-stromal tumour	25	16	8/4/8/4	8/8	8/8	8/8
Fibroma	13	31	15/8/15/8	15/15	15/15	15/15
Thecoma	2	0	0/0/0/0	0/0	0/0	0/0
Granulosa cell tumour	10	0	0/0/0/0	0/0	0/0	0/0
Germ cell tumour	32	56	31/44/38/3	25/31	50/6	56/13
Mature teratoma	15	67	13/47/47/0	40/27	67/0	67/0
Immature teratoma	3	100	100/100/100/0	0/100	67/33	100/100
Struma ovarii	10	40	40/30/10/0	20/20	40/0	40/0
York sac tumour	3	0	0/0/0/0	0/0	0/0	0/0
Dysgerminoma	1	100	100/100/100/100	0/100	0/100	100/100
Metastasis	13	0	0/0/0/0	0/0	0/0	0/0
Colon cancer	5	0	0/0/0/0	0/0	0/0	0/0
Pancreatic cancer	2	0	0/0/0/0	0/0	0/0	0/0
Gastric cancer	2	0	0/0/0/0	0/0	0/0	0/0
Appendiceal cancer, breast cancer, uterine cancer, malignant lymphoma	4	0	0/0/0/0	0/0	0/0	0/0

BLT, borderline tumour

**Table 3:** The number of cases, frequency of calcification, frequency of each calcification pattern, number of calcifications, and their distribution and localisation across epithelial tumours

	Cases (n)	Calcification (%)	Punctate/Linear/Coarse/Amorphous (%)	Few/Many (%)	Focal/Diffuse (%)	Cyst wall or septa/Solid part (%)
Epithelial tumour	258	35	27/15/5/10	22/13	30/5	28/9
Serous tumour	85	26	16/7/4/16	15/11	16/9	12/18
Serous benign tumour	10	20	10/0/0/10	20/0	20/0	20/0
Serous BLT	7	29	29/0/0/14	29/0	29/0	29/0
LGSC	2	100	100/100/50/50	0/100	0/100	100/50
HGSC	66	24	14/6/3/17	14/11	15/9	6/21
Mucinous tumour	70	49	44/31/6/6	26/23	43/6	46/3
Mucinous benign tumour	24	71	63/50/4/0	42/ 29	63/8	67/0
Mucinous BLT	26	31	31/23/8/8	15/19	27/8	35/0
Mucinous carcinoma	20	45	40/20/5/10	25/20	40/5	35/10
Clear cell tumour	57	28	19/12/4/7	21/7	28/0	26/5
Clear cell carcinoma	57	28	19/12/4/7	21/7	28/0	26/5
Endometrioid tumour	29	31	24/14/7/0	24/7	28/3	31/0
Endometrioid BLT	4	50	50/25/25/ 0	25/25	50/0	50/0
Endometrioid carcinoma	25	28	20/12/4/0	24/4	24/4	28/0
Seromucinous tumour	12	50	42/0/8/8	50/0	50/0	50/0
Seromucinous benign tumour	1	100	0/0/0/100	100 /0	100 /0	100/0
Seromucinous BLT	7	14	14/0/0/0	14/0	14/0	14/0
Seromucinous carcinoma	4	100	100/0/23/0	100/0	100/0	100/0
Brenner tumour	5	100	20/0/0/80	20/80	80/20	20/80
Brenner tumour (Benign)	5	100	20/0/0/80	20/80	80/20	20/80

BLT, borderline tumour; LGSC, low-grade serous carcinoma; HGSC, high-grade serous carcinoma

Differences in the frequency of calcifications were observed among the major tumour categories (*p* < 0.001), namely epithelial tumours, sex cord-stromal tumours, germ cell tumours, and metastatic tumours, with calcifications being more common in germ cell tumours and less common in metastatic tumours. Significant differences in the frequency of calcifications were also observed among the histological subtypes of epithelial tumours (*p* = 0.005). Mucinous and Brenner tumours showed a higher prevalence of calcification than the other histological types, while serous tumours exhibited a lower prevalence of calcification than the other histological types. A significant difference in the frequency of calcifications was also observed when comparing the malignancy grades (benign tumour, borderline tumour, and carcinoma) across all histological types of epithelial tumours (*p* < 0.001), benign tumours exhibited a higher prevalence of calcification than borderline tumours and carcinomas. Among the non-epithelial tumours, none of the granulosa cell or metastatic tumours exhibited calcification.

When comparing the calcification patterns observed among the major tumour categories, significant differences were found for all calcification patterns: punctate (*p* = 0.024), linear (*p* < 0.001), coarse (*p* < 0.001), and amorphous (*p* < 0.001). Punctate and coarse calcifications were more frequently observed in epithelial and germ cell tumours compared to other types, linear calcifications were more common in germ cell tumours, and amorphous calcifications were predominantly seen in epithelial tumours. Immature teratomas often showed a mixture of punctate, linear, and coarse calcifications. When comparing the calcification patterns observed among the histological subtypes of epithelial tumours, significant differences were found for punctate (*p* = 0.002), linear (*p* = 0.001), and amorphous (*p* < 0.001). Punctate calcifications were more frequently observed in serous and mucinous tumours, linear calcifications were more common in mucinous tumours, and amorphous calcifications were more prevalent in serous tumours and Brenner tumours, compared to other types of epithelial tumours. In clear cell tumours, endometrioid tumours, and mucinous tumours, cases with a single punctate calcification were common.

In the analysis of the number, distribution, and localisation of calcifications, germ cell tumours were found to frequently exhibit a higher number of calcifications compared to other tumour categories. Among epithelial tumours, mucinous tumours and Brenner tumours demonstrated a similar tendency. Regarding the distribution and localisation, amorphous calcifications were diffusely scattered within the solid tissues, whereas linear calcifications were predominantly identified in the walls and septa. In mucinous tumours with abundant calcifications, these were generally diffusely distributed throughout. Meanwhile, coarse calcifications in mature teratomas were typically observed within the wall or localized to the Rokitansky protuberance.

The agreement of the three readers indicated that the presence of calcification showed substantial agreement, with a κ-value of 0.68. For each calcification pattern, κ-values ranged from 0.24 to 0.77, with lower agreement for punctate (κ = 0.27) and amorphous (κ = 0.24) patterns, and higher agreement for linear (κ = 0.77) and coarse (κ = 0.72) patterns. The evaluation of the number of calcifications demonstrated moderate agreement, with a κ-value of 0.45. In contrast, the distribution of calcifications showed fair agreement, with a κ-value of 0.27. Lastly, the assessment of localization of calcifications showed moderate agreement, with a κ-value of 0.42.

**Fig. 3:**
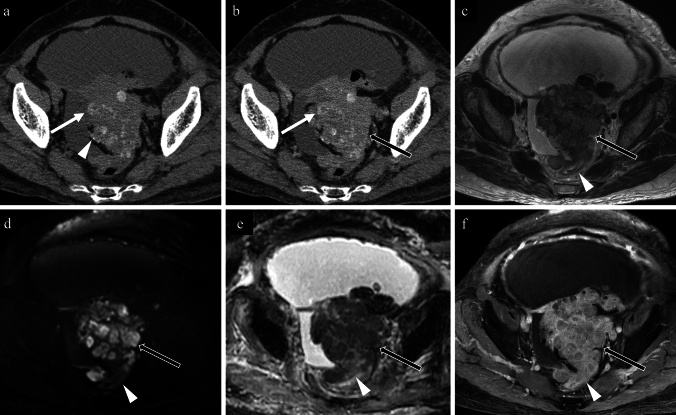
73-year-old woman with a high-grade serous carcinoma of the left fallopian tube. **a** non-contrast CT; **b** contrast-enhanced CT; **c** axial T2-weighted image; **d** axial diffusion-weighted image; **e** axial apparent diffusion coefficient map; **f** axial contrast-enhanced T1-weighted image. A lobulated mass in the sigmoid colon fossa is observed, accompanied by diffuse amorphous calcifications (**a**; white arrow) and punctate calcifications (**a**; white arrowhead) on non-contrast CT. Contrast-enhanced CT shows faint enhancement of the entire mass (**b**; black arrow), and it is difficult to determine the presence of calcification only on contrast-enhanced CT (**b**; white arrow). T2-weighted image demonstrates heterogeneous signal intensities (**c**; black arrow). Diffusion-weighted image shows strong but heterogeneous diffusion restriction (**b** and **e**; black arrows). Postcontrast T1-weighted image shows moderate enhancement (**f**; black arrow). Infiltration from the rectum is also observed (**c**–**f**; arrowheads). Pathological findings confirmed the diagnosis of high-grade serous carcinoma originating from the left fallopian tube

**Fig. 4:**
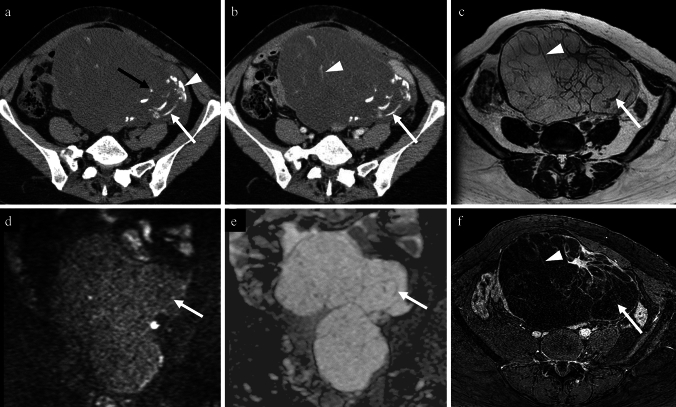
49-year-old woman with a mucinous cystadenoma of the left ovary. **a** non-contrast CT; **b** contrast-enhanced CT; **c** axial T2-weighted image; **d** coronal diffusion-weighted image; **e** coronal apparent diffusion coefficient map; **f** axial contrast-enhanced T1-weighted image. A cystic tumour containing multiple septa is observed, with predominant linear calcifications aligned with the septa (a; white arrow), accompanied by areas of punctate (**a**; black arrow) and coarse calcifications (**a**; white arrowhead) on non-contrast-CT. Contrast-enhanced CT revealed enhancement along the septa (**b**, arrowhead), however the enhancement effect of the calcified septa is unclear (**b**, arrow). On T2-weighted image, the cystic content appears relatively homogeneous, and the calcified septa (**c**; arrow) is slightly thicker than the non-calcified ones (**c**; arrowhead). No diffusion restriction is noted on the diffusion-weighted image (**d** and **e**; arrows). The post-contrast T1-weighted image shows smooth enhancement along the septa (**f**; arrowhead), but the enhancement effect of the calcified septa is not evident (**f**; arrow). Pathological examination revealed a mucinous cystadenoma

**Fig. 5:**
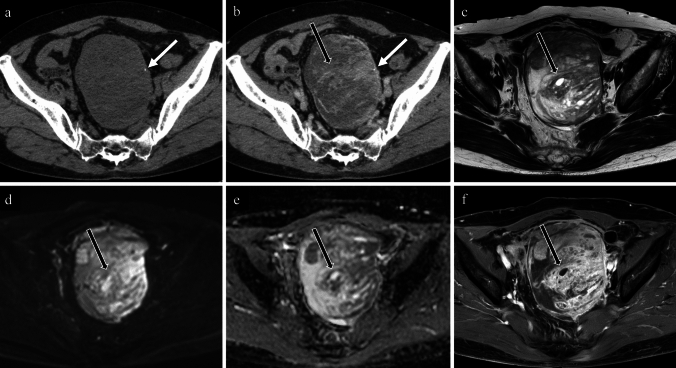
67-year-old woman with a clear cell carcinoma of the left ovary. **a** non-contrast CT; **b** contrast-enhanced CT; **c** axial T2-weighted image; **d** axial diffusion-weighted image; **e** axial apparent diffusion coefficient map; **f** axial contrast-enhanced T1-weighted image. Non-contrast CT reveals a single punctate calcification in the left wall of the cystic tumour (**a**; white arrow). On contrast-enhanced CT, the papillary solid tissues are well-enhanced (**b**; black arrows), making the calcification less prominent (**b**; white arrow). On T2-weighted imaging, a unilocular cystic lesion with tall, papillary solid tissues is observed, and the solid tissues show intermediate signal intensities and contain small internal cystic components (**c**; black arrow). Strong diffusion restriction and significant enhancement are observed in the solid tissues (**d**–**f**; black arrows). Pathological examination confirmed a clear cell carcinoma associated with endometriosis

**Fig. 6:**
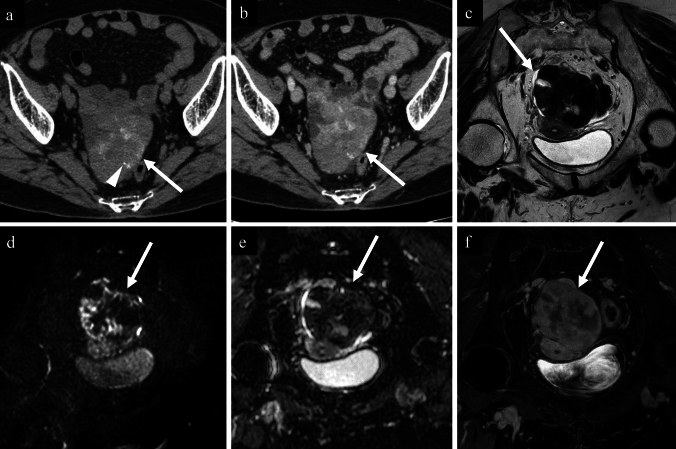
77-year-old woman with a Brenner tumour of the right ovary. **a** non-contrast CT; **b** contrast-enhanced CT; **c** coronal T2-weighted image; **d** coronal diffusion-weighted image; **e** coronal apparent diffusion coefficient map; **f** coronal contrast-enhanced T1-weighted image. A lobulated tumour with amorphous calcifications (**a**; arrow) and punctate calcifications (**a**; white arrowhead) is observed in the right ovary. Contrast-enhanced CT shows weak enhancement throughout the tumour (**b**; arrow), making the identification of calcification difficult on contrast-enhanced CT alone. This solid mass is heterogeneous but markedly low signals on T2-weighted image (c; arrow) with poor diffusion restriction on diffusion-weighted image (**d** and **e**; arrows). After contrast administration, enhancement is predominant at the periphery (**f**; arrow). The tumour was excised and diagnosed as a Brenner tumour

**Fig. 7:**
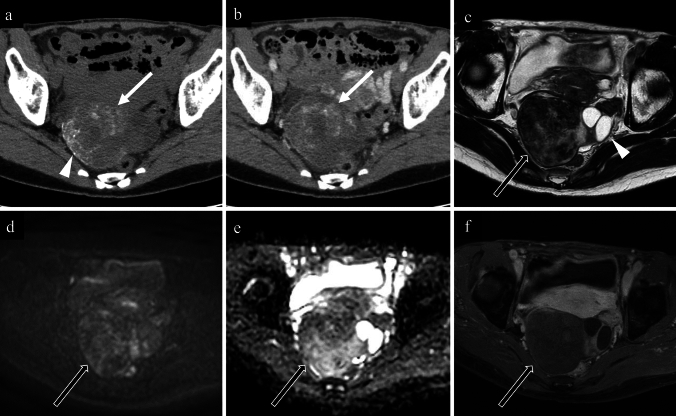
65-year-old woman with a fibroma of the right ovary. **a** non-contrast CT; **b** contrast-enhanced CT; **c** axial T2-weighted image; **d** axial diffusion-weighted image; **e** axial apparent diffusion coefficient map; **f** axial contrast-enhanced T1-weighted image. A round mass is observed on the dorsal side of the uterus, accompanied by amorphous (a; arrow) and punctate calcifications (a; arrowhead). The enhancement is poor, and the contrast-enhanced CT cannot distinguish between the amorphous calcifications and enhancement effects. On T2-weighted imaging, the mass appears as a markedly low-signal solid tumour (a; black arrow) with cysts (c; arrowhead) on its left margin. It shows weak diffusion restriction (c and d; black arrows) and poor enhancement (f; black arrow), consistent with a fibroma. Pathologically, it was diagnosed as a fibroma. The calcification patterns of fibromas varied, and this was the only case exhibiting amorphous calcifications

**Fig. 8:**
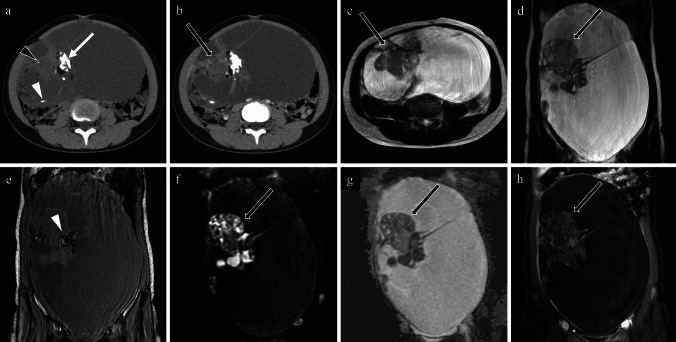
18-year-old woman with an immature teratoma of the left ovary. **a** non-contrast CT; **b** contrast-enhanced CT; **c** axial T2-weighted image; **d** coronal T2-weighted image; **e** coronal T1-weighted image (opposed-phase chemical shift image); **f** coronal diffusion-weighted image; **g** coronal apparent diffusion coefficient map; **h** coronal contrast-enhanced T1-weighted image. A large cystic tumour occupies the abdominal cavity. Coarse calcifications (**a**; white arrow) and fat are observed at the margins of the solid tissue. Punctate (a; black arrowhead) and linear (**a**; white arrowhead) calcifications are also present. Post-contrast enhancement corresponds to the solid tissue (**b**, black arrow). On T2-weighted image, the solid tissue appears with moderate signal intensities (**c** and **d**; black arrows). In the opposite phase of the chemical shift T1-weighted image, the fat component shows signal reduction (**e**, white arrowhead). On the diffusion-weighted image, the solid tissue exhibits heterogeneity and strong diffusion restriction (**f** and **g**, black arrows), while the post-contrast T1-weighted image shows heterogeneous enhancement (**h**, black arrow). The tumour was pathologically diagnosed as an immature teratoma

Figures 3, 4, 5, 6, 7, 8 show non-contrast CT, contrast-enhanced CT, T2-weighted, diffusion-weighted, apparent diffusion coefficient (ADC) map, and fat-saturated contrast-enhanced T1-weighted MR images of high-grade serous carcinoma, mucinous adenoma, clear cell carcinoma, Brenner tumour, fibroma, and immature teratoma, each accompanied by calcifications.

## Discussion

Mucinous and Brenner tumours had a significantly higher frequency of calcification, whereas serous tumours had a significantly lower frequency of calcification. Comparing the malignancy grades across all types of epithelial tumours, benign tumours had a significantly higher frequency of calcification. Regarding calcification patterns, the amorphous calcification was significantly more common in serous and Brenner tumours, whereas the linear type was significantly more frequent in mucinous tumours.

The differentiation of histological types of ovarian tumours has primarily been performed using MRI [3, 4], with little attention given to CT. Although it is well known that ovarian tumours frequently exhibit calcifications, no studies have comprehensively examined the frequency or histological variations of these calcifications specifically using non-contrast CT. Heterotopic calcification, defined as the deposition of calcium salts in abnormal sites, is traditionally classified into two types: dystrophic and metastatic. Dystrophic calcification occurs in dying tissues with normal serum calcium levels, while metastatic calcification results from disturbances in calcium metabolism, often leading to hypercalcemia and calcium accumulation influenced by tumour cells or the tumour microenvironment [3]. However, Silva et al.'s study challenges these traditional binary classifications. Their findings indicate that ovarian calcifications, primarily located in the stroma, are driven by hormonal stimulation and associated with metabolic changes in stromal cells. This secretory aetiology differs from classical dystrophic and metastatic calcifications, possibly representing a novel phenomenon [14]. Qing Wang et al. investigated non-contrast or contrast-enhanced CT of ovarian lesions in 222 patients aged ≤ 20 years. Normal ovarian cysts appeared with calcification in the wall in 12/32 cases, demonstrating that calcification in normal ovaries is frequently observed even in young patients, and this finding also further suggesting a link to hormones [4]. Psammoma bodies, corresponding to amorphous calcifications in this study, are concentric calcified structures commonly found in papillary thyroid carcinoma, meningioma, and serous carcinoma of the ovary, but are rarely found in other tumours [15]. Although initially thought to result from dystrophic calcification [3], studies on ovaries and meningiomas have suggested that collagen production by neoplastic cells and subsequent calcification form psammoma bodies [4, 5]. Furthermore, in meningiomas and papillary thyroid carcinoma, precursor forms of psammoma bodies—extracellular hyaline globules surrounded by neoplastic cells—have been reported, which lack the surrounding cellular degeneration typically associated with psammoma bodies [6, 7]. Therefore, psammoma bodies are now recognized as a result of active biological processes involving secretion rather than passive tissue degeneration.

In epithelial tumours, several studies encompassing a large number of ovarian tumours assessed calcification using CT. Burkill et al. investigated 1,721 patients with ovarian cancer and found that 8% had calcification, however, the presence of calcification was based on reported records and examined primarily using contrast-enhanced CT [5]. The frequency of calcification in serous tumours, including those evaluated with contrast-enhanced CT, has been reported to vary widely, ranging from 5 to 60% [5, 13, 14]. Pathological reports in serous tumours also show a wide variation, with frequencies ranging from 14 to 73% [4, 14]. Regarding the patterns of calcifications, a report indicated that punctate calcification pattern was common in serous cystadenomas, similar to our findings [4]. The pathogenesis of low-grade serous carcinoma is believed to follow a stepwise progression from cystadenoma to atypical proliferative serous tumour, to non-invasive micropapillary serous borderline tumour, and eventually to low-grade serous carcinoma. Typical psammoma bodies are reported to occur frequently in serous tumours, particularly in low-grade serous carcinoma, with a prevalence of 50–90% [15, 16]. The high frequency of calcifications observed in low-grade serous carcinoma in this study, despite the small sample size, was consistent with previous findings. The frequency of calcification in mucinous tumours, including those evaluated with contrast-enhanced CT, has been reported to range from 11 to 34% [5, 13, 14]. One pathological report in mucinous tumours, indicated a high frequency of 57% [6], and similar to our results, the frequency of calcification in mucinous tumours on CT has been reported to be higher than in serous tumours [6]. Calcifications in mucinous tumours are reported to occur in two locations: within the wall and inside the cyst. The first type consists of laminar and coarse deposits in fibrotic and hyalinised dense connective tissue and is often found in the walls or septa of benign mucinous tumours, suggesting secretory calcification, and these are not observed in serous tumours [14, 16]. The second type is found in the necrotic material of proliferating tumours: dystrophic calcification [6]. In the current study, mucinous tumours often showed linear calcifications along the walls and septa, consistent with the former type of calcification. The frequency of calcification in the solid tissues of Brenner tumours, evaluated by non-contrast or contrast-enhanced CT, has been reported to be as high as 56% to 80%, similar to our findings [7, 17]. Montoriola et al. classified these calcifications as punctate in 89% of cases and coalescent in 11%. Referring to the images they presented, this punctate calcification also includes what we refer to as amorphous calcification [8]. Simons et al. histopathologically analysed mucinous tumours associated with Brenner tumours and with teratomas. They found calcifications in 56% of the mucinous tumours associated with Brenner tumours and 22% of those associated with teratomas, indicating a higher frequency in the former [18]. However, in the current study, tumours mixed with other components were excluded, therefore, Brenner tumours with mucinous components were also excluded. Pathologically, Brenner tumours characteristically demonstrate extensive amorphous calcification within the solid tissues, and calcification has been reported in the fibrous stroma [19].

In sex cord-stromal tumours, Pat et al. reviewed the contrast-enhanced CT findings of fibrothecomas and reported calcifications in 7% [9]. Histopathological evaluations also showed that the frequency of calcification in fibrothecomas was relatively low, with 15% in fibromas and 22% to 29% in thecomas [2, 20]. Although there were no relevant cases in the current study, it is known that ovarian fibromas associated with nevoid basal cell carcinoma syndrome are known to frequently show calcification compared to ovarian fibromas not associated with nevoid basal cell carcinoma [21]. Calcification in fibrothecomas is known to occur within the fibrous stroma of the tumour. Although there are no studies on the mechanism by which calcification occurs, fibrothecoma is known as a hormone-producing tumour, and the fact that calcification is more common in young patients [2, 20] and those with genetic diseases [21] suggests secretory calcification. No other reports existed of calcification in granulosa cell tumours on CT scans, while several reports of histopathological calcification did exist, and the frequency of calcification was low, with only 2 out of 16 cases [20] and 1 out of 15 cases showing calcification [22].

Regarding germ cell tumours, Qing Wang et al. investigated ovarian lesions in patients aged < 20 years using non-contrast or contrast-enhanced CT, and 97% of mature teratoma presented with coarse calcification. All seven cases of immature teratomas presented with fine calcification [4]. Nakamori et al. evaluated the non-contrast CT scans of teratomas in patients aged < 20 years and reported that the number of calcifications in immature teratomas was significantly higher than that in mature teratomas. Moreover, the maximum diameter of the calcifications of immature teratomas was significantly larger than that of mature teratomas because the immature teratomas showed scattered calcifications of variable size [11], which is consistent with our findings of mixed calcifications in immature teratomas. Calcification of teratomas corresponds to bone, cartilage, and teeth, and the calcification of immature teratomas is usually associated with solid components. Ikeuchi et al. reported that in non-contrast or contrast-enhanced CT scans of struma ovarii cases, curvilinear calcifications were observed in the thickened septa or cyst walls in 54% of cases [10]. In our study, the frequency of calcification in struma ovarii was also high, with the second most common calcification pattern being linear. In gonadoblastomas, punctate calcifications were reported to be frequently observed on CT scans [23] and could even be identifiable on plain radiographs because of their coarse appearance [24].

With regard to metastasis, none of the metastatic tumours in our case showed calcification; however, there are several case reports of ovarian metastasis from colorectal cancer in which calcification was observed on CT scans [25]. Although our cases did not include such instances, ovarian metastasis is presumed to be more likely to present with calcification in primary tumours that are prone to calcification, such as bone tumours, including osteosarcoma, and thyroid papillary carcinoma.

Regarding the relationship between the malignancy grade and calcification, Burkill et al. classified tumours into borderline malignancy and ovarian cancer grades 1–3, noting that the calcified group tended to have tumours of significantly lower grades [5]. Silva et al. showed that calcification in serous tumours was correlated with a lower histological grade and may indicate a poorer survival rate [14]. Also, Motohara et al. revealed the same result in serous tumours [26]. Similar results were obtained in the present study, in which benign epithelial tumours had a significantly higher frequency of calcification. However, this may reflect the influence of mucinous tumours, which had the largest number of benign cases, rather than serous tumours.

This study has several limitations. First, the number of cases with relatively rare histological types was small. Second, because CT scans were typically performed only in cases where malignancy or complications were suspected, the prevalence of benign tumours, such as mature teratomas, was much lower than their general incidence rate. Third, high-grade serous carcinoma was treated as part of the same group as other serous tumours, but currently, high-grade serous carcinoma and other serous tumours are considered entirely different tumours. Forth, as indicated by the low interobserver agreement, determining the presence of amorphous calcification and distinguishing it from punctate calcification is not always straightforward. Fifth, calcification at the margins or the presence of a single calcification was considered indicative of calcification. Therefore, normal ovarian calcification may be present [27]. Finally, a pathological evaluation of calcification was not performed, and the sensitivity of CT for detecting calcification is known to be lower than that for detecting pathological calcification [6]. However, adding a non-contrast pelvic CT scan solely for the evaluation of calcification is not clinically essential, and virtual non-contrast images generated by dual-energy CT may serve as a useful alternative [28].

In conclusion, after evaluating the non-contrast CT scans of 328 patients, mucinous and Brenner tumours showed a significantly higher frequency of calcification. In contrast, serous tumours had a significantly lower frequency of calcification. Benign tumours showed more frequent calcifications across all epithelial types. Additionally, the amorphous calcification pattern was more common in serous and Brenner tumours whereas the linear type was significantly more frequent in mucinous tumour. It is also likely that most calcifications in ovarian tumours can be classified as secretory calcifications. These findings highlight the distinct calcification profiles that can aid in the diagnosis and characterisation of ovarian tumours in radiological practice.

## Data Availability

The datasets used and analysed during the current study are available from the corresponding author on reasonable request.
